# Real-World Antibiotic Strategies for Multidrug-Resistant *Pseudomonas aeruginosa*: Evidence from the Italian SUSANA Multicentre Study

**DOI:** 10.3390/antibiotics15080796

**Published:** 2026-08-17

**Authors:** Francesca Gavaruzzi, Giordano Madeddu, Elena Delfina Ricci, Matteo Faltoni, Alessandra Bandera, Francesca Colucci, Andrea De Vito, Angelo Maccaro, Paolo Maggi, Stefania Piconi, Giovanni Cenderello, Marco Merli, Leonardo Luzi, Giuseppe Vittorio De Socio, Silvia Mascolo, Linda Bussini, Goffredo Angioni, Paolo Bonfanti, Stefania Cicalini

**Affiliations:** 1 Systemic and Immune Depression-Associated Infections Unit, National Institute for Infectious Diseases Lazzaro Spallanzani, Istituto di Ricovero e Cura a Carattere Scientifico, 00149 Rome, Italy; francesca.gavaruzzi@inmi.it; 2 Unit of Infectious Diseases, Department of Medicine, Surgery, and Pharmacy, University of Sassari, 07100 Sassari, Italy; giordano@uniss.it (G.M.); andrea.devito@aouss.it (A.D.V.); 3 Fondazione ASIA Onlus, Buccinasco, 20900 Milan, Italy; ed.ricci@libero.it; 4 Fondazione San Gerardo dei Tintori, Istituto di Ricovero e Cura a Carattere Scientifico, University of Milano-Bicocca, 20900 Monza, Italy; matteo.faltoni@irccs-sangerardo.it (M.F.); paolo.bonfanti@unimib.it (P.B.); 5 Fondazione Ca’ Granda Ospedale Maggiore Policlinico, Istituto di Ricovero e Cura a Carattere Scientifico, 20122 Milan, Italy; alessandra.bandera@policlinico.mi.it (A.B.); angelo.maccaro@policlinico.mi.it (A.M.); 6 Azienda Ospedaliera di Rilievo Nazionale Sant’Anna e San Sebastiano, 81100 Caserta, Italy; francesca.colucci@aorncaserta.it; 7 Department of Medicine and Surgery, Kore University of Enna, 94100 Enna, Italy; paolo.maggi@unikore.it; 8 Alessandro Manzoni Hospital, 23900 Lecco, Italy; s.piconi@asst-lecco.it; 9 Sanremo Hospital, 18038 Sanremo, Italy; g.cenderello@asl1.liguria.it; 10 Niguarda Hospital, 20162 Milan, Italy; marco.merli@ospedaleniguarda.it (M.M.); l.luzi1@campus.unimib.it (L.L.); 11 Infectious Disease Unit, Department of Medicine and Surgery, Santa Maria Hospital, University of Perugia, 06156 Perugia, Italy; giuseppe.desocio@unipg.it; 12 Cotugno Hospital, Azienda Ospedaliera dei Colli, 80131 Naples, Italy; silvia.mascolo@ospedalideicolli.it; 13 Humanitas Research Hospital, Istituto di Ricovero e Cura a Carattere Scientifico, Rozzano, 20089 Milan, Italy; linda.bussini@hunimed.eu; 14 Santissima Trinità Hospital, 09121 Cagliari, Italy; goffredo.angioni@aslcagliari.it

**Keywords:** *Pseudomonas aeruginosa*, multidrug-resistant, pneumonia, bloodstream infection, ceftolozane/tazobactam, ceftazidime/avibactam, combination therapy, prolonged infusion, continuous infusion

## Abstract

**Background/Objectives:** Multidrug-resistant (MDR) *Pseudomonas aeruginosa* pneumonia and bloodstream infections (BSI) remain challenging because of their severity, limited therapeutic options, and high mortality, despite the availability of novel β-lactam-based agents. We aimed to describe real-world treatment patterns, characteristics associated with treatment and infusion-strategy selection, and the association of these strategies with clinical outcomes in the retrospective, multicentre Italian SUSANA cohort, an observational study evaluating the use, safety, and outcomes of novel antibiotics in hospitalized patients. **Methods:** Adults with MDR *P. aeruginosa* pneumonia and/or BSI treated with novel antipseudomonal agents for ≥72 h were included. Treatment was classified as monotherapy or combination therapy, and infusion strategy as prolonged/continuous or standard. The primary outcome was 28-day all-cause mortality. A multivariable Cox regression analysis was performed. **Results:** Among 173 patients, pneumonia was the most frequent clinical presentation (74.0%). Monotherapy was used in 111 patients (64.2%), combination therapy in 62 (35.8%), and prolonged/continuous infusion in 120 (69.4%). Ceftolozane–tazobactam (CT) (64.7%) and ceftazidime–avibactam (CZA) (20.2%) were the most prescribed agents. Patients receiving prolonged/continuous infusion, regardless of monotherapy or combination therapy, had greater baseline clinical severity than those receiving standard infusion. Overall, 28-day mortality was 23.7%. In the multivariable analysis, neither combination therapy nor prolonged/continuous infusion was independently associated with reduced mortality, whereas septic shock (aHR 4.10, 95% CI 1.01–16.66) and ICU admission (aHR 2.53, 95% CI 1.06–6.08) increased mortality risk. **Conclusions:** In this multicentre real-world cohort, treatment strategies for MDR *P. aeruginosa* pneumonia and BSI appeared to be influenced by baseline severity and patient-related factors. Mortality was more strongly associated with markers of severe illness, including ICU admission and septic shock. No independent association with improved survival was observed for combination therapy compared with monotherapy or for prolonged/continuous compared with standard infusion. These findings highlight the prognostic role of baseline clinical severity and support the need for prospective, randomized, pharmacokinetic/pharmacodynamic (PK/PD)-guided studies to identify patients who may benefit from these strategies.

## 1. Introduction

Infections caused by multidrug-resistant (MDR) *Pseudomonas aeruginosa* represent a major challenge in contemporary clinical practice, particularly among hospitalized and critically ill patients. *P. aeruginosa* is characterized by intrinsic resistance to multiple antimicrobial classes and by a remarkable ability to acquire additional resistance mechanisms, resulting in limited therapeutic options and poor clinical outcomes, especially in pneumonia and bloodstream infections (BSI) [1,2]. Observational studies have shown that infections caused by MDR *P. aeruginosa* are associated with mortality rates approaching 40–50%, prolonged hospital stays, and increased resource utilization, especially in patients admitted to the intensive care unit (ICU) [3]. Among these syndromes, hospital-acquired pneumonia (HAP), ventilator-associated pneumonia (VAP), and BSI are particularly relevant because they frequently occur in critically ill patients and are associated with high bacterial burden, difficult source control, microbiological persistence, recurrence, and poor outcomes [2,3].

In recent years, the development of novel β-lactam/β-lactamase inhibitor (BL/BLI) combinations, including ceftolozane–tazobactam (CT), ceftazidime–avibactam (CZA) and imipenem–relebactam (IR), as well as the siderophore cephalosporin cefiderocol, has significantly expanded the therapeutic armamentarium against MDR Gram-negative infections [2,4]. These agents have shown favorable in vitro activity and clinical efficacy against *P. aeruginosa*, particularly in the absence of metallo-β-lactamases and are now recommended as preferred options in international treatment guidelines [5].

However, the clinical effectiveness of β-lactam agents depends not only on antimicrobial activity but also on achieving adequate pharmacokinetic/pharmacodynamic targets at the site of infection. This issue is particularly relevant in critically ill patients, in whom sepsis, septic shock, augmented renal clearance, renal replacement therapy, extracorporeal support, hypoalbuminemia, and increased volume of distribution may substantially alter β-lactam exposure [6,7]. Since β-lactams exhibit time-dependent antibacterial activity, prolonged or continuous infusion strategies have been proposed to maximize the percentage of time that free drug concentrations remain above the minimum inhibitory concentration, especially in infections caused by less susceptible isolates or in difficult-to-penetrate sites such as the lung [6,7,8,9]. In this context, optimized infusion strategies may improve target attainment, limiting underexposure, and potentially reducing microbiological failure or emergence of resistance [5,6,9,10].

Despite the availability of these novel antipseudomonal agents, the optimal therapeutic strategy for MDR *P. aeruginosa* infections remains uncertain. Combination therapy is frequently adopted in clinical practice, especially in patients with severe illness, with the theoretical aim of improving early treatment adequacy, achieving synergistic antibacterial activity, or preventing the emergence of resistance [10]. However, supporting evidence for the routine use of combination therapy as definitive treatment is limited. Studies conducted with older antipseudomonal β-lactams failed to demonstrate a consistent survival benefit of combination regimens compared with monotherapy, while reporting increased toxicity, particularly nephrotoxicity [10,11].

Moreover, combination therapy is often preferentially prescribed to patients with greater clinical severity, septic shock, or a higher risk of treatment failure [12,13]. Consequently, apparent differences in outcomes between treatment groups may be affected by confounding by indication rather than reflecting the effect of the treatment strategy itself [12,14].

Importantly, most currently available studies have primarily focused on treatment outcomes, whereas comparative real-world evidence on how novel antipseudomonal agents are administered remains limited. In particular, few contemporary studies have simultaneously evaluated monotherapy versus combination therapy, standard versus prolonged or continuous infusion, and the clinical factors associated with the selection of these strategies. Evidence is especially scarce for pneumonia and BSI caused by MDR *P. aeruginosa* in multicentre European cohorts, and the extent to which baseline severity influences treatment allocation and observed outcomes remains insufficiently characterized [15].

To address these gaps, we conducted a multicentre observational study within the Italian SUSANA cohort, a retrospective study evaluating the use, safety, and outcomes of novel antibiotics in hospitalized patients. We aimed to investigate antibiotic treatment and infusion strategies for MDR *P. aeruginosa* pneumonia and BSI, describe the patient and treatment characteristics associated with their selection, and assess their association with 28-day mortality.

## 2. Results

### 2.1. Study Population

**Figure 1:**
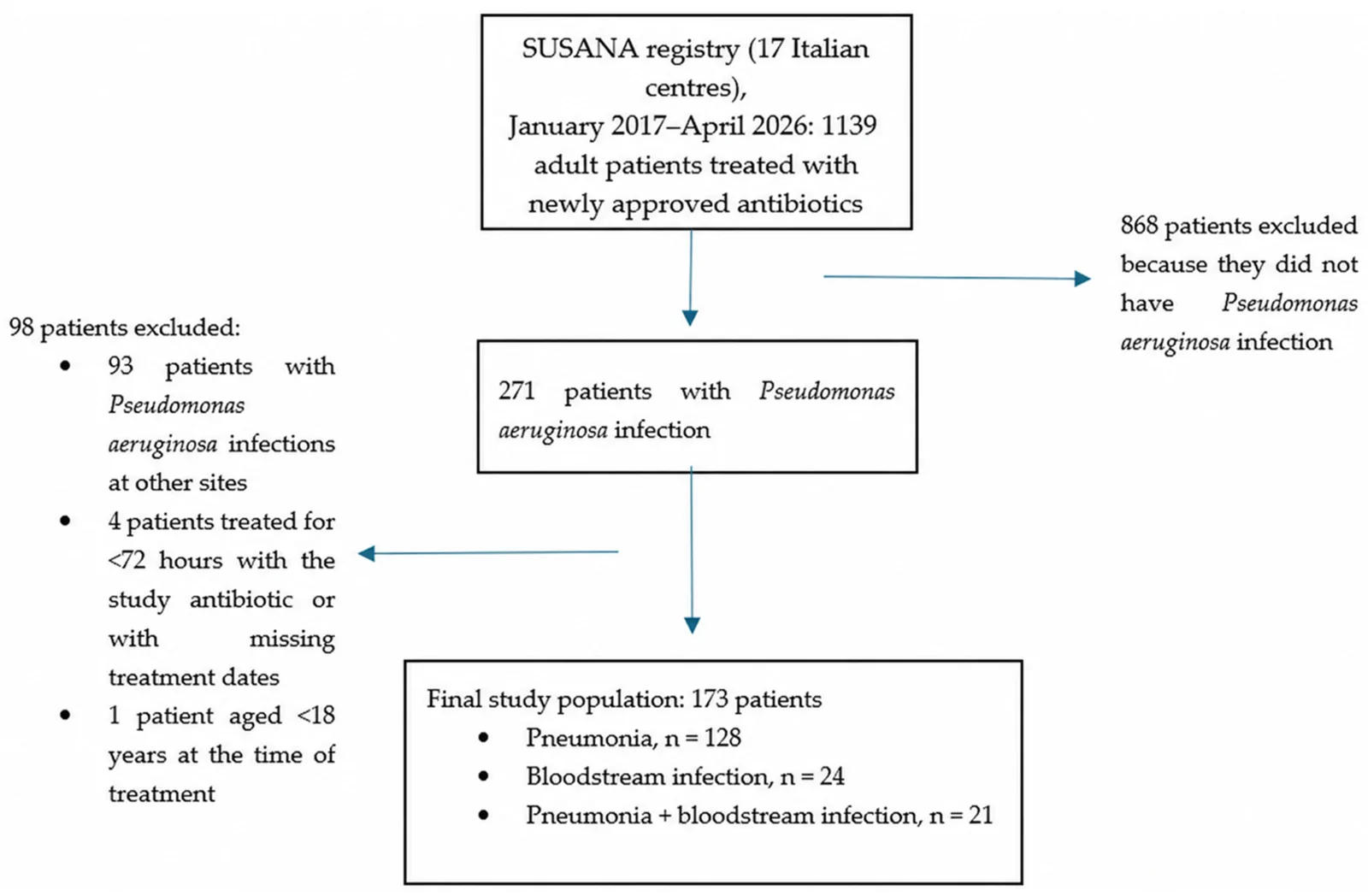
Flowchart of study population selection.

Among 1139 patients enrolled in the SUSANA study, 271 had *P. aeruginosa* infection. After exclusion of infections from other sites, treatment duration < 72 h, missing treatment dates, and age < 18 years, 173 patients were included in the final analysis (Figure 1). Most infections were pneumonia (128/173, 74.0%), followed by BSI (24/173, 13.9%) and combined pneumonia plus BSI (21/173, 12.1%). Overall, monotherapy was administered in 111/173 patients (64.2%), whereas 62/173 (35.8%) received combination therapy. The most frequently used agents were CT (64.7%) and CZA (20.2%). Regarding infusion strategy, prolonged or continuous infusion was used in 120/173 patients (69.4%), whereas standard intermittent infusion was used in 53/173 patients (30.6%). In a sensitivity analysis, inclusion of the two excluded patients with a known treatment duration of <72 h did not materially change the main findings.

### 2.2. Baseline Characteristics According to Treatment Strategy

Patients receiving monotherapy were significantly older than those treated with combination therapy (median age 68 vs. 63 years, *p* = 0.008) and more frequently treated with antibiotics in the previous 90 days (86.5% vs. 50.0%, *p* < 0.0001 (Table 1). Monotherapy was also associated with higher rates of sepsis or septic shock (78.4% vs. 62.9%, *p* = 0.028). Likewise, when infusion strategy was classified as prolonged/continuous versus standard infusion, a significant difference was observed between monotherapy and combination therapy groups (76.6% vs. 56.4% for prolonged/continuous infusion; *p* = 0.0059). No significant differences were observed between treatment groups regarding infection type. However, differences were observed in the distribution of β-lactam-based agents, with CT being more frequently administered as monotherapy (75.7% vs. 45.2%), whereas CZA was more commonly used in combination regimens (7.2% vs. 43.6%; *p* < 0.0001). These substantial baseline and treatment-allocation differences indicate that the choice between monotherapy and combination therapy was not random and may have been influenced by clinical severity, prior antimicrobial exposure, respiratory support, and the selected β-lactam agent, suggesting potential confounding by indication. No significant differences were observed between treatment groups in clinical cure, microbiological recurrence or 28-day mortality.

### 2.3. Baseline Characteristics According to Infusion Strategy

Patients receiving prolonged or continuous infusion showed a more severe clinical profile than those receiving standard intermittent infusion as detailed in Supplementary Table S1, which provides the complete comparison of demographic characteristics, comorbidities, previous healthcare exposures, invasive devices, infection characteristics, antimicrobial treatment, and clinical outcomes according to infusion strategy. ICU admission at infection onset was more frequent in the prolonged/continuous infusion group (68.3% vs. 47.2%, *p* = 0.0083), and the distribution of sepsis severity differed significantly between groups, with sepsis or septic shock being more common among patients receiving prolonged/continuous infusion (*p* < 0.0001). These Supplementary Materials support the presence of important baseline imbalances between infusion groups and the possibility of confounding by indication.

Infection type was similarly distributed between groups, with pneumonia being the most common presentation in both prolonged/continuous and standard infusion groups (74.2% vs. 73.6%; *p* = 0.2776). Treatment allocation significantly differed according to infusion strategy: monotherapy was used in 70.8% of patients receiving prolonged/continuous infusion and in 49.1% of those receiving standard infusion, while combination therapy was used in 29.2% and 50.9%, respectively (*p* = 0.0059). No significant differences were observed in treatment duration, clinical cure, microbiological recurrence, or 28-day mortality between groups. Twenty-eight-day mortality was 25.8% in the prolonged/continuous infusion group and 18.9% in the standard infusion group (log-rank *p* = 0.3152).

### 2.4. Survival Analysis and Predictors of 28-Day Mortality

Overall, 41/173 (23.7%) patients died within 28 days. In univariable Cox regression, combination therapy was not associated with a lower 28-day mortality compared with monotherapy (HR 0.789, 95% CI 0.409–1.523; *p* = 0.480). When infusion strategy was analysed as prolonged/continuous versus standard intermittent infusion, prolonged/continuous infusion was not associated with a significant reduction in 28-day mortality in univariable analysis (HR 1.436, 95% CI 0.704–2.929; *p* = 0.320) (Table 2). All variables reported in Table 1 were screened for their potential association with 28-day mortality, based on clinical relevance, and their potential role as confounders. If the association had a p-value lower than 0.20, the variables were included in the multivariable Cox regression model. Treatment strategy and infusion modality were retained regardless of their univariable statistical significance, as they represented the main exposures of interest.

Treatment duration was significantly associated with 28-day mortality, with HR 0.869 by 1 day more (95% CI 0.806–0.937). Since shorter duration may reflect early death, we performed two models, with and without the variable (Table 3).

**Figure 2:**
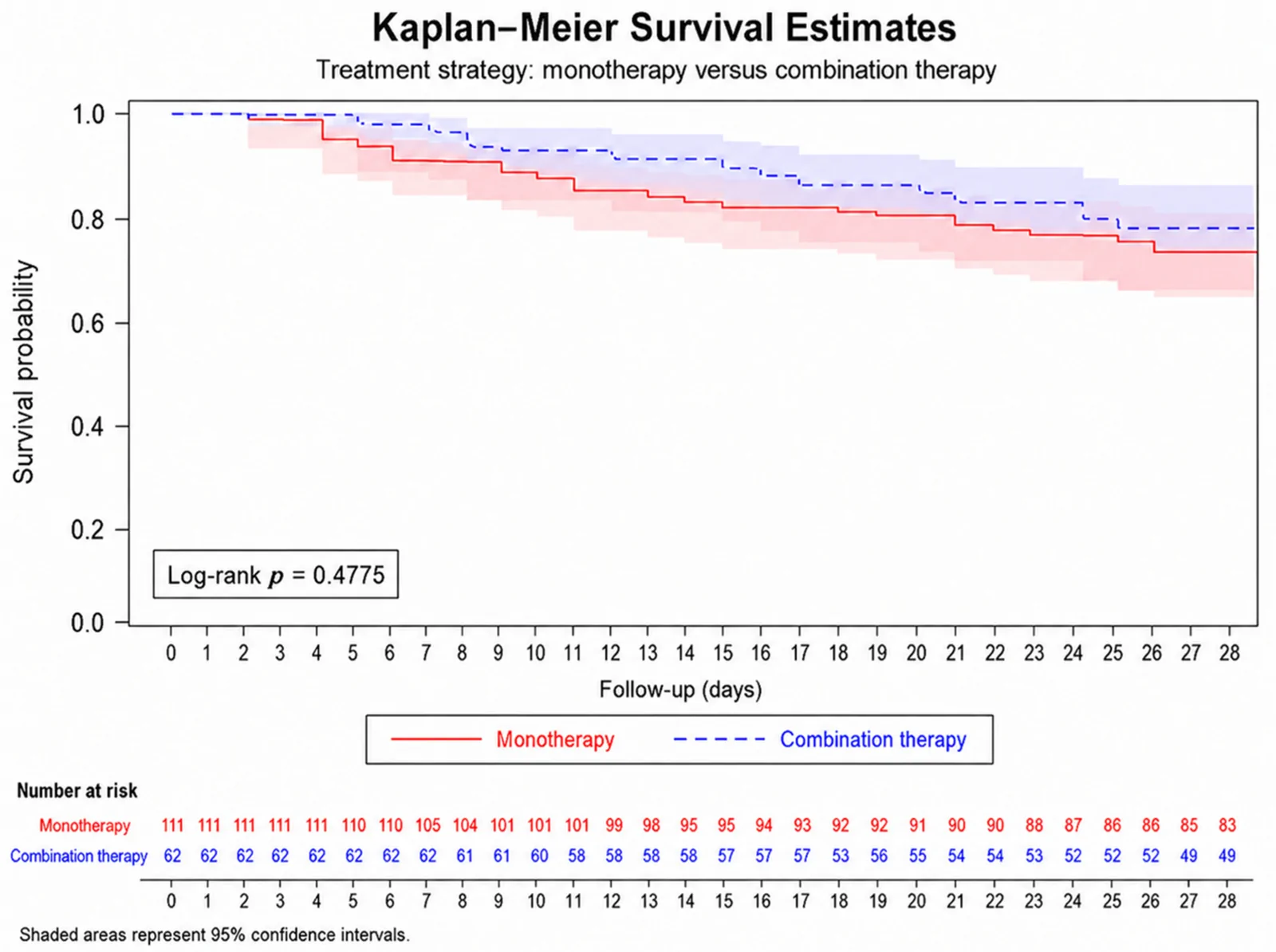
Kaplan–Meier curve on 28-day survival according to treatment strategy: monotherapy versus combination therapy.

**Figure 3:**
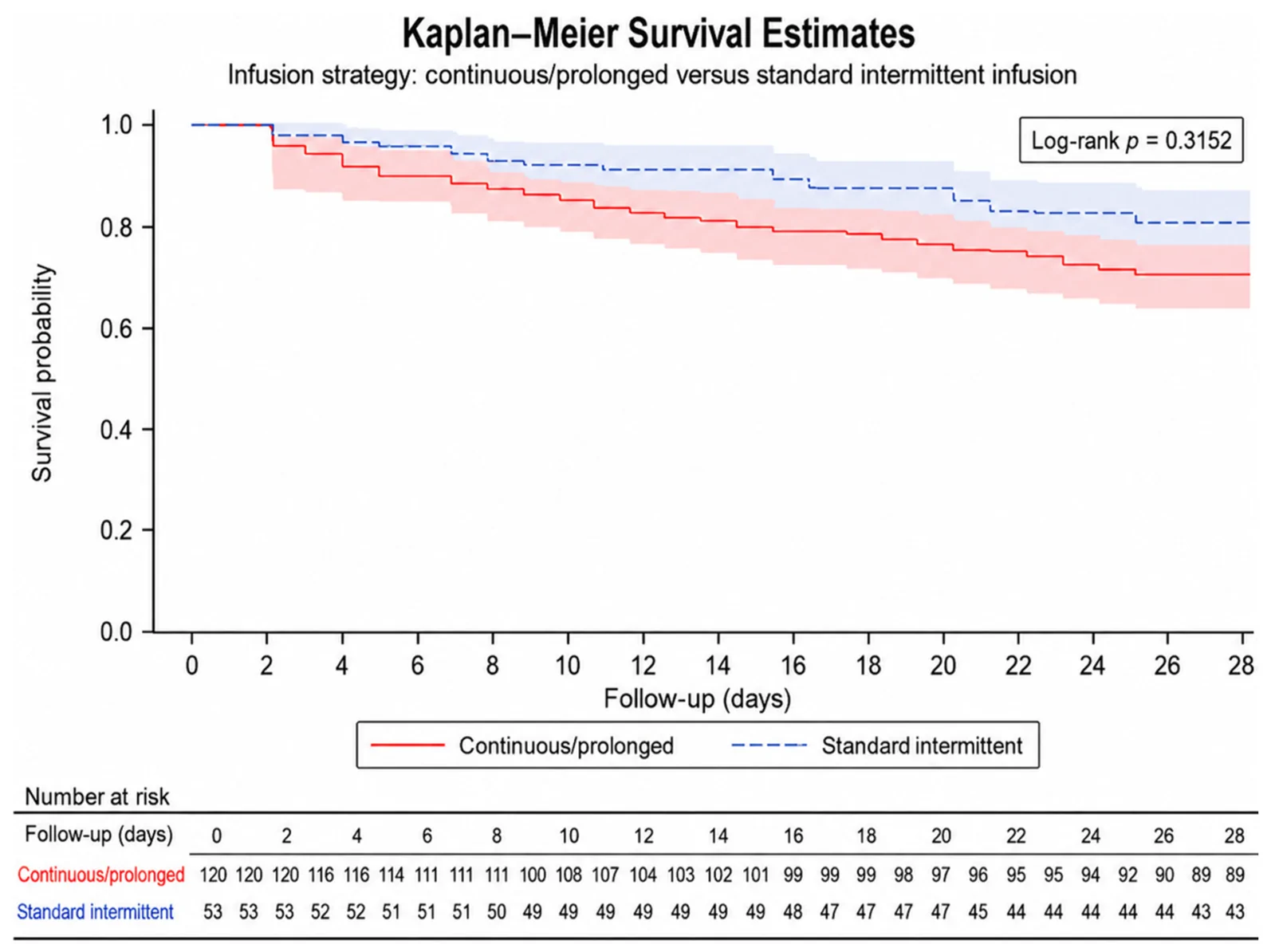
Kaplan–Meier curve on 28-day survival according to infusion strategy: prolonged/continuous versus standard intermittent infusion.

The Kaplan–Meier curves showed no significant difference in the probability of 28-day survival between patients receiving monotherapy and those receiving combination therapy (Figure 2). Similarly, no significant survival difference was observed between prolonged/continuous and standard intermittent infusion strategies (Figure 3).

### 2.5. Exploratory Subgroup Analyses

Given the limited sample sizes and number of outcome events, all subgroup analyses were considered exploratory and were not powered to establish definitive treatment effects. Their findings should therefore be interpreted as hypothesis-generating.

Exploratory subgroup analyses were performed among patients with septic shock and among those with VAP. In the septic shock subgroup, no significant differences in clinical outcomes or 28-day mortality were observed according to treatment or infusion strategy.

Among 42 patients with severe VAP, 30/42 (71.4%) received prolonged/continuous infusion and 12/42 (28.6%) received standard intermittent infusion. Clinical cure was similar between groups (53.3% vs. 50.0%), while 28-day mortality was numerically lower in the prolonged/continuous infusion group, although the difference was not statistically significant (20.0% vs. 41.7%; Fisher *p* = 0.2432). In univariable Cox regression, prolonged/continuous infusion was associated with a non-significant trend toward lower 28-day mortality compared with standard infusion (HR 0.44, 95% CI 0.13–1.43; *p* = 0.170).

Because of the small subgroup size, the limited number of deaths, and the wide confidence interval, this numerical difference should not be interpreted as evidence of a survival benefit.

## 3. Discussion

In this multicentre real-world cohort of patients with multidrug-resistant *P. aeruginosa* pneumonia and BSI treated with novel β-lactam-based agents, we evaluated whether treatment strategy, infusion modality, and choice of antimicrobial agent were associated with clinical outcomes. Overall, neither combination therapy nor prolonged/continuous infusion was independently associated with improved 28-day survival.

These findings are consistent with previous observational studies that failed to demonstrate a clear survival advantage of definitive combination therapy compared with active β-lactam monotherapy [11,12], and with the inconsistent evidence regarding the impact of optimized β-lactam infusion strategies on mortality [8,16,17].

Mortality appeared to be driven primarily by baseline clinical severity, especially ICU admission and concomitant sepsis/septic shock, rather than by the treatment strategies assessed. Nonetheless, the absence of an independent association does not imply therapeutic equivalence nor exclude a causal effect of treatment. Instead, any true treatment effect may have been difficult to detect because of underlying differences in disease severity, non-random treatment allocation, and residual confounding.

A relevant finding is that combination therapy was not simply used in patients with higher global severity. Patients receiving monotherapy were older, more comorbid, and more frequently had chronic kidney disease (CKD), ICU admission, and septic shock. Conversely, combination therapy was more often used in younger patients with better renal function, and was also associated with respiratory/airway devices, suggesting a respiratory or ventilatory phenotype rather than a simple “more severe–more combination therapy” pattern. These findings suggest that treatment selection may have reflected different dimensions of clinical complexity. Renal dysfunction, frailty, and toxicity concerns may have discouraged adjunctive agents, whereas mechanical ventilation, high respiratory bacterial burden, or concern for microbiological persistence may have favoured combination treatment. Notably, infusion strategy differed significantly between treatment groups. Prolonged or continuous infusion was more frequently used in patients receiving monotherapy, whereas standard infusion was more common in the combination therapy group. This suggests that treatment strategy and infusion modality may have reflected different real-world prescribing approaches rather than independent decisions. In some cases, clinicians may have chosen to optimize β-lactam exposure through prolonged or continuous infusion instead of adding a second active agent. Accordingly, combination therapy and infusion modality were partly interrelated exposures, which further limits causal interpretation of their individual effects.

Polymicrobial infection did not appear to explain this choice, as polymicrobial episodes occurred in both monotherapy and combination therapy groups (18.9% vs. 12.9%). Among polymicrobial infections, *Klebsiella pneumoniae* was the most frequent co-isolated microorganism, followed by *Enterococcus faecium* and *Serratia marcescens.* Supplementary Table S2 provides the detailed distribution of co-isolated microorganisms according to the novel β-lactam administered, supporting the interpretation of agent-specific prescribing patterns in polymicrobial infections. The overall frequency of polymicrobial infections across treatment groups suggests that the addition of further agents was not primarily driven by the need to cover co-pathogens, although the microbiological context may have influenced selection of individual β-lactams. The lack of a significant mortality benefit with combination therapy may have several explanations. First, when an active novel β-lactam-based agent is available, the incremental benefit of adding a second antimicrobial may be limited, although this hypothesis cannot be confirmed from our observational data. While combination therapy has a strong theoretical rationale in Gram-negative infections, including broader coverage, potential synergy, and prevention of resistance emergence, these advantages have not consistently translated into improved survival when an active β-lactam is used as definitive therapy [10]. This is consistent with data from *P. aeruginosa* BSI showing no clear advantage of definitive combination therapy over monotherapy [11] and with more recent real-world evidence on novel β-lactam/β-lactamase inhibitors in drug-resistant *P. aeruginosa*, where combination regimens did not show a consistent clinical benefit [18]. Second, toxicity concerns may have influenced treatment allocation. In our cohort, combination therapy most commonly consisted of one additional active antimicrobial, particularly fosfomycin, aminoglycosides, or colistin, while nearly one-third of patients in the combination therapy group received two or more additional active agents. The choice of adjunctive therapy may have been influenced by patient characteristics, as concerns regarding toxicity, drug interactions, or overall treatment burden could have discouraged the use of certain combination regimens in older, frailer patients or in those with chronic kidney disease or septic shock. This heterogeneity should be considered when interpreting the lack of survival benefit, as “combination therapy” encompassed different regimens with potentially distinct pharmacological rationales, safety profiles, and clinical effects. Therefore, the numerically higher crude mortality observed in the monotherapy group may primarily reflect baseline frailty and severity rather than a detrimental effect of monotherapy itself. Third, combination therapy seemed to be preferentially selected for a respiratory or ventilatory phenotype, whereas prolonged or continuous infusion was more frequently adopted in patients treated with monotherapy, suggesting different clinician-driven strategies for antimicrobial optimization. The higher frequency of respiratory/airway devices in the combination group suggests that clinicians may have been more likely to add a second agent in patients with ventilated pneumonia, where high bacterial burden, airway colonization, biofilm, impaired lung penetration, and microbiological persistence are major concerns. This interpretation is consistent with recent expert reviews on MDR *P. aeruginosa*, which support combination therapy only in selected scenarios rather than as a routine definitive strategy [19]. Nevertheless, these prescribing patterns cannot establish that either strategy was preferable or more effective in a specific clinical phenotype.

From a clinical perspective, our findings do not support the routine addition of a second active antimicrobial once susceptibility to an appropriate novel antipseudomonal β-lactam has been confirmed, in line with current recommendations [5,19,20]. However, the study cannot exclude a role for combination therapy in selected situations, such as uncertain susceptibility, limited source control, high bacterial burden, or concern for resistance emergence.

Although C/T and CZA were the most frequently prescribed agents, alternative novel β-lactam options within our cohort included cefiderocol, IR, and M/V, whose selection may have been influenced by local susceptibility profiles, infection characteristics, and drug availability. Beyond these agents, alternative therapeutic options for MDR *P. aeruginosa* may include aminoglycosides, polymyxins, or fosfomycin-based combination regimens, particularly when susceptibility to newer β-lactams is not documented or active agents are not available, although their use is often limited by toxicity concerns and less favorable pharmacokinetic and clinical profiles. The preferential use of targeted novel agents may also reflect antimicrobial stewardship efforts aimed at reducing exposure to more toxic rescue therapies and limiting unnecessary combination treatment, which could otherwise increase selective pressure without providing clear additional benefit.

Polymicrobial infections were not more frequent in the combination group overall, but their distribution differed across individual agents, with C/T mainly used in monomicrobial infections and CZA, cefiderocol, and M/V proportionally more represented among polymicrobial episodes. M/V was used in only nine patients, eight from a single centre, suggesting a centre-specific prescribing pattern. More than half of these episodes were polymicrobial, mainly involving *K. pneumoniae*, which may partly explain its selection in real-world practice. However, the activity of M/V against MDR *P. aeruginosa* is limited and highly dependent on the underlying resistance mechanism, with no predictable added benefit of vaborbactam over meropenem in most resistance phenotypes. Therefore, in the absence of systematic M/V MICs or resistance mechanism data, the microbiological rationale for its use in these cases cannot be precisely inferred [21,22,23].

Infection sites should also be considered when interpreting molecule-specific outcomes. Pneumonia represented the predominant infection site in our cohort and is a challenging MDR *P. aeruginosa* phenotype, due to high bacterial burden, difficult airway source control, limited lung penetration, airway colonization, and microbiological persistence. Recent comparative data suggest that outcomes may differ according to both agent and infection site, and the CACTUS study provides one of the few real-world head-to-head comparisons between C/T and CZA in MDR *P. aeruginosa* [24]. Similarly, recent reviews highlight the lack of robust head-to-head evidence among newer agents and support individualized treatment selection based on susceptibility patterns, resistance mechanisms, infection site, and local experience [16,18]. Therefore, the absence of significant outcome differences in our cohort should not be interpreted as equivalence among all drugs or clinical situations.

The use of prolonged or continuous infusion was frequent in our cohort, involving almost 70% of patients. This likely reflects the growing adoption of PK/PD-oriented prescribing strategies for severe MDR *P. aeruginosa* infections. Patients receiving prolonged/continuous infusion had a more severe clinical profile, with higher rates of ICU admission, sepsis or septic shock, and invasive devices. However, despite this higher baseline complexity, prolonged/continuous infusion was not independently associated with improved 28-day survival. In the multivariable Cox model excluding treatment duration, prolonged/continuous infusion showed no significant association with mortality compared with standard infusion (aHR 0.918, 95% CI 0.424–1.987; *p* = 0.827). These results should not be interpreted as evidence of equivalence between the two infusion strategies, but rather as indicating that no independent association could be detected within this cohort.

This finding should be interpreted in light of the strong biological rationale and the heterogeneous clinical evidence supporting optimized β-lactam infusion. Since β-lactams exhibit time-dependent activity, prolonged or continuous infusion may improve the probability of maintaining free drug concentrations above the MIC, particularly in critically ill patients with altered pharmacokinetics, high bacterial burden, augmented renal clearance, renal replacement therapy, or difficult-to-penetrate sites such as the lung [6,7,8]. For CT specifically, continuous infusion has been associated with higher probability of PK/PD target attainment in patients infected with *P. aeruginosa* [9].

Nevertheless, clinical studies have not uniformly demonstrated a mortality benefit, probably due to the heterogenous samples in terms of clinical severity, infectious site and antimicrobial susceptibility of isolates. Recent meta-analytic evidence suggests that prolonged β-lactam infusion may reduce mortality in critically ill patients with sepsis or septic shock [25]. Observational studies have reported favourable outcomes in specific settings, including Gram-negative BSI treated with extended-infusion β-lactams [26], BSI in patients with liver cirrhosis treated with piperacillin/tazobactam or carbapenems [27], KPC-producing *K. pneumoniae* infections treated with CZA [28], and HAP/VAP treated with CZA, with or without fosfomycin, within the SUSANA cohort [29]. In contrast, large, randomized trials provided less definitive results; the BLING III study did not show a statistically significant reduction in 90-day mortality with the continuous infusion of piperacillin/tazobactam or meropenem in critically ill patients with sepsis, although clinical cure was higher. The MERCY study found no improvement with the continuous meropenem in critically ill patients with sepsis or septic shock [16,17].

Thus, our findings do not argue against PK/PD optimization but rather highlight the difficulty of demonstrating its independent impact on mortality in retrospective real-world cohorts. In our study, prolonged/continuous infusion may have acted as a marker of clinician-perceived PK/PD risk, since it was preferentially selected in patients considered at higher risk of underexposure or treatment failure. Moreover, the absence of systematic data on exact antibiotic dosing, renal dose adjustment, MIC values for each administered agent, and the mechanisms underlying the MDR phenotype limit our ability to determine whether prolonged/continuous infusion has achieved adequate PK/PD target attainment. Therefore, infusion modality should be regarded as a surrogate for an exposure-optimization strategy rather than as a direct measure of adequate antibiotic exposure.

Overall, 28-day mortality in our cohort was 23.7%, which appears lower than mortality rates reported in some previous cohorts of resistant *P. aeruginosa* infections, particularly BSI and ICU populations [3,30].

This may reflect the contemporary use of novel β-lactam-based agents, the heterogeneous distribution of infection sites, the relatively limited proportion of patients with septic shock at baseline, and the exclusion of patients treated for less than 72 h, which may have reduced the representation of very early treatment failures. However, comparisons across studies should remain cautious because of differences in populations, infection severity, inclusion criteria, and treatment exposure. No significant differences were observed between monotherapy and combination therapy, or between prolonged/continuous and standard infusion, in terms of clinical cure, microbiological recurrence, or 28-day mortality. The numerical trend toward higher mortality and treatment failure in the monotherapy group may reflect the higher baseline severity and frailty of these patients rather than a detrimental effect of monotherapy itself. The longer treatment duration observed in the combination group should also be interpreted cautiously. It may reflect more prolonged courses in complex respiratory infections, whereas the shorter duration in the monotherapy group may partly result from early death or treatment interruption among patients with greater baseline severity. Thus, treatment duration should be considered a post-baseline variable potentially affected by survival time itself, and should not be interpreted as a direct marker of treatment efficacy. For this reason, we performed a second multivariable model excluding treatment duration. No adverse events attributable to the administered novel β-lactam-based agents were recorded, although this finding should be interpreted cautiously because safety assessment was based on retrospective data collection.

Finally, microbiological recurrence and resistance emergence are difficult to evaluate retrospectively without standardized microbiological follow-up, systematic MIC reassessment, or resistance mechanism data. This is particularly relevant in MDR *P. aeruginosa*, where treatment failure may be driven not only by inadequate clinical response but also by the selection of resistant subpopulations during therapy. Previous work on C/T resistance has suggested that optimized exposure may be relevant for limiting resistance emergence, while broader reviews on ESKAPE pathogens emphasize the role of antimicrobial pressure and adaptive resistance mechanisms in recurrent or persistent infections [31].

Exploratory subgroup analyses were performed in patients with septic shock and in those with severe VAP. No significant differences were observed in the septic shock subgroup.

Given the limited sample size and the small number of outcome events, these findings cannot rule out clinically meaningful differences between treatment strategies. Accordingly, they should not be interpreted as indicating that outcomes were driven solely by host-related factors or the degree of organ dysfunction.

Among patients with severe VAP, prolonged/continuous infusion was associated with numerically lower 28-day mortality compared with standard infusion, although this difference did not reach statistical significance. Because the subgroup included only 42 patients, with 12 receiving standard infusion, and the confidence interval was wide, this numerical difference should be regarded solely as hypothesis-generating and not as evidence of a survival benefit. Although a possible benefit remains biologically plausible, given the challenges of drug exposure, lung penetration, high bacterial burden, and microbiological persistence in VAP [6,8,9], the present analysis cannot determine whether the observed numerical difference reflects a true effect of the infusion strategy or is attributable to chance, residual confounding, or other unmeasured clinical factors. This signal is also consistent with the concept that pneumonia due to MDR *P. aeruginosa* may behave differently from BSI alone. Recent comparative data suggest that outcomes with newer agents may vary according to infection site, with pneumonia representing a particularly challenging phenotype [24]. Similarly, recent real-world data on IR in MDR *P. aeruginosa* pneumonia and BSI highlighted the importance of late outcomes, including recurrence and emergence of resistance, beyond early mortality alone [32]. These observations support further investigation but do not provide confirmatory evidence for a site-specific treatment effect in our cohort.

This study has several limitations. First, its retrospective observational design exposes the analysis to residual confounding and confounding by indication; propensity scores were also substantially different between comparison groups; thus, matching would have further lowered the sample size. Treatment strategy, choice of agent, and infusion modality were selected by treating physicians and may have been influenced by unmeasured factors, including local practice, drug availability, perceived severity, and microbiological uncertainty. Moreover, potential centre-level differences in prescribing practices could not be formally accounted for, as the limited number of outcomes precluded reliable adjustment for centre effects. Although participating centres generally operate according to contemporary standards of care, residual between-centre heterogeneity cannot be excluded. Although multivariable adjustment accounted for measured differences, it could not fully reproduce the counterfactual comparison between strategies. Second, detailed pharmacological and microbiological data were incomplete. Exact antibiotic dosing, dose reductions according to renal function, and dose adjustments during renal replacement therapy were not systematically available. Similarly, MIC values for each administered agent and the mechanisms underlying the MDR phenotype of *P. aeruginosa* were not consistently collected. Therapeutic drug monitoring was also unavailable. These limitations reduce our ability to correlate infusion strategy with actual drug exposure, susceptibility profile, resistance mechanisms, and clinical outcomes. Third, the time interval between clinical onset and initiation of an active antibacterial regimen was not systematically captured. Delays in active therapy may have influenced patient outcomes and could not be accounted for in the analysis. Fourth, the number of patients receiving less commonly used agents, including cefiderocol, IR, and M/V, was small, limiting molecule-specific analyses. Finally, subgroup analyses, particularly those in septic shock and severe VAP, were exploratory and underpowered because of the limited number of patients and events.

Despite these limitations, our study has several strengths. It represents a contemporary multicentre real-world cohort focused specifically on MDR *P. aeruginosa* pneumonia and BSI treated with novel β-lactam-based agents. Unlike many previous studies focusing on a single drug, a single infection site, or only monotherapy versus combination therapy, our analysis simultaneously evaluated treatment strategy, infusion modality, drug selection, and 28-day mortality, providing insight into real-world prescribing patterns in complex MDR *P. aeruginosa* infections.

Future prospective studies, ideally randomized controlled trials, are needed to clarify the role of combination therapy and optimized β-lactam infusion in MDR *P. aeruginosa* infections. These studies should incorporate PK/PD-guided approaches, therapeutic drug monitoring, microbiological and resistance data, and standardized definitions of treatment strategies and outcomes. Stratification by infection site, disease severity, and renal function may help identify patient subgroups most likely to benefit. Beyond mortality, relevant endpoints should include clinical and microbiological response, recurrence, resistance emergence, toxicity, and antibiotic exposure metrics.

## 4. Materials and Methods

### 4.1. Study Design and Setting

We performed a retrospective, observational, multicentre cohort study within the Italian SUSANA network (SUrveillance of SAfety and outcome of New Antibiotics), an ongoing national project collecting real-world data on the use, effectiveness, and safety of newly approved antimicrobial agents across Italian infectious diseases units. For the present analysis, we included centres reporting cases of MDR *Pseudomonas aeruginosa* pneumonia and/or BSI treated with novel antipseudomonal agents. The study period ranged from January 2017 to April 2026 according to the most recent data available in the SUSANA database; the database was queried with a data cut-off in May 2026. Seventeen Italian centres participated in the SUSANA project and contributed eligible cases to the present analysis.

### 4.2. Study Population

Adult patients (≥18 years) were eligible if they had a microbiologically documented MDR *P. aeruginosa* infection, defined according the Infectious Diseases Society of America guidance and the European Society of Clinical Microbiology and Infection guidelines as not susceptible to at least one antibiotic in at last three antibiotic classes to which *P. aeruginosa* is generally expected to be susceptible: penicillins, cephalosporins, fluroquinolones, aminoglycosides, and carbapenems [5,20], based on local microbiology reports and susceptibility testing available in the SUSANA database, together with a clinical diagnosis of pneumonia (HAP, VAP) and/or BSI. Patients were required to receive treatment with a novel antipseudomonal antibiotic for at least 72 h. Episodes considered colonization, cases lacking key microbiological or outcome data, and recurrent infectious episodes in the same patient were excluded; only the first episode was included in the analysis. Polymicrobial infection was defined as the isolation of one or more clinically relevant microorganisms in addition to *P. aeruginosa* during the same infectious episode. Polymicrobial episodes were not excluded from the analysis; co-isolated microorganisms were recorded and described.

### 4.3. Antimicrobial Treatment Strategies

Patients received C/T, CZA, cefiderocol, IR, or M/V, according to real-world prescribing practice. M/V was included when prescribed by treating physicians, although molecule-specific interpretation was limited by the absence of systematic MIC values and resistance mechanism characterization. Treatment strategy was categorized as monotherapy when the novel agent was administered without other concomitant systemic antibacterials, and as combination therapy when the novel agent was administered together with at least one additional antipseudomonal agent with expected antipseudomonal activity for ≥48 h of overlap. Combination regimens could include aminoglycosides, polymyxins, fluoroquinolones, fosfomycin, aztreonam, or carbapenems, according to local clinical practice and physician judgment. Because a substantial proportion of episodes were polymicrobial, concomitant agents prescribed exclusively to target co-isolated non-*Pseudomonas* pathogens (e.g., anti-Gram-positive coverage) were recorded as concomitant therapy but were not considered part of the antipseudomonal “combination therapy” definition. For β-lactam agents, infusion strategy was categorized as intermittent infusion (infusion duration ≤ 1 h), prolonged/extended infusion (infusion duration ≥ 3 h), or continuous infusion (24 h infusion). This categorization was based on infusion duration as recorded in the SUSANA database and aligned with definitions commonly used in comparative studies of extended-infusion β-lactam therapy. Choice of antimicrobial regimen, infusion modality and treatment duration was left to the discretion of treating physicians.

### 4.4. Data Collection and Definitions

Demographic characteristics, comorbidities, Charlson Comorbidity Index (CCI), previous hospitalization and antibiotic exposure, infection type, ICU admission, treatment variables, invasive devices, and clinical outcomes were extracted from the SUSANA electronic database. Cardiovascular disease included heart failure, previous myocardial infarction, peripheral vascular disease, and prior stroke/transient ischemic attack. CKD was defined as moderate-to-severe CKD or dialysis dependence. Immunosuppression included steroid therapy, chemotherapy, immunotherapy, monoclonal antibodies, neutropenia, AIDS, and transplant-related immunosuppressive regimens. Invasive devices were grouped into predefined categories, including vascular devices (central venous catheters, PICC lines, and port-a-cath devices), urinary catheters, respiratory/airway devices (endotracheal tubes and tracheostomy cannulas), nutritional-enteral devices (feeding tubes and gastrostomy devices), and drainage/derivation devices (biliary or chest drainage, external ventricular drainage, and ventriculoperitoneal shunts). Advanced extracorporeal support devices, including extracorporeal membrane oxygenation (ECMO) and continuous venovenous hemofiltration (CVVH), were recorded separately. Pneumonia was defined as a new lung infiltrate plus clinical evidence of infection (e.g., new onset of fever, purulent respiratory secretions, leukocytosis, or worsening oxygenation). HAP was defined as pneumonia not incubating at hospital admission and occurring ≥48 h after hospital admission; VAP was defined as pneumonia occurring >48 h after endotracheal intubation. BSI was defined as at least one positive blood culture for *P. aeruginosa* in the context of clinical infection. Sepsis and septic shock were defined according to Sepsis-3 criteria. Sepsis was defined as life-threatening organ dysfunction secondary to infection, while septic shock was defined as sepsis requiring vasopressor support to maintain mean arterial pressure ≥ 65 mmHg and serum lactate > 2 mmol/L despite adequate fluid resuscitation.

### 4.5. Outcomes

The primary outcome was all-cause 28-day mortality from initiation of novel antipseudomonal therapy. Secondary outcomes included clinical cure and microbiological recurrence. Clinical cure was defined as resolution of signs and symptoms related to infection at the end of treatment without need for additional antipseudomonal therapy. Microbiological recurrence was defined as isolation of the same microorganism from the same anatomical site within 30 days from the first isolate. Clinical cure, microbiological recurrence, and death were evaluated as mutually exclusive outcomes among the 168 patients with available outcome data, after excluding five patients who discontinued empirical therapy. Secondary outcomes also included describing patient and treatment characteristics associated with the selection of monotherapy versus combination therapy and with infusion strategy.

### 4.6. Statistical Analysis

Continuous variables were expressed as median and interquartile range (IQR), while categorical variables were reported as counts and percentages. Comparisons between groups were performed using Mann–Whitney U test or Kruskal–Wallis test for continuous variables, as appropriate, and χ^2^ test or Fisher’s exact test for categorical variables.

Missing data management was predefined according to variable type and the extent of missing data. If a Y/N variable was missing for fewer than five people, we assumed it was ‘N’. If it was missing for five or more people, we created a ‘missing’ class to be included in the survival analysis. No continuous variable had missing values. If age (for none of the cases) or the number of days of treatment (for four cases) could not be retrieved after querying the centre, the case was excluded. The standardized difference in means or proportions was reported as the difference between means or proportions divided by their pooled standard error. Imbalance was defined as an absolute value greater than 0.20 (small effect size). For skewed continuous variables, we used rank means.

Overall survival was evaluated considering 28-day mortality as the primary outcome. Survival curves were estimated using the Kaplan–Meier method and compared using the log-rank test. Factors associated with 28-day mortality were evaluated using Cox proportional hazards regression models. The proportional hazards assumption was evaluated visually by plotting the log of the negative log of the estimated survival functions against time, and by plotting the negative log of the estimated survival functions against time (i.e., the Kaplan–Meier curve). The interaction between the predictors and the log-time function was also tested in the multivariate model by testing all the retained variables at once.

Variables with *p* < 0.20 in univariable analyses and variables considered clinically relevant were included in multivariable models. Hazard ratios (HRs) and adjusted hazard ratios (aHRs) were reported with 95% confidence intervals (95% CI). Given the exploratory nature of the study and the limited number of events, multivariable models were kept parsimonious to reduce the risk of overfitting. The CCI and the single comorbidities were used alternatively in multivariable models. Similarly, a strong correlation is likely present between sepsis and ICU admission; thus, we created a variable including both information, and alternatively included it in the models. Treatment duration was interpreted cautiously because of potential survivorship/reverse causality bias, as patients who died early inevitably received shorter treatment courses. Thus, we performed two models, with and without the variable. No formal sample size calculation was performed because of the retrospective exploratory design. All tests were two-sided and *p* values < 0.05 were considered statistically significant. Statistical analyses were performed using SAS software version 9.4 (SAS Institute, Cary, NC, USA).

## 5. Conclusions

In conclusion, in this multicentre real-world cohort of MDR *P. aeruginosa* pneumonia and BSI, neither combination therapy nor prolonged/continuous infusion was independently associated with reduced 28-day mortality. Treatment choices appeared to reflect clinical phenotype, baseline severity, renal function, toxicity concerns, infection site, and PK/PD-oriented prescribing rather than acting as isolated determinants of outcome. These findings support an individualized approach to combination therapy and optimized infusion strategies, reserving them for selected high-risk scenarios rather than applying them routinely to all patients. Prospective studies incorporating MIC data, resistance mechanisms, exact dosing, therapeutic drug monitoring, and standardized PK/PD targets are needed to identify which patients are most likely to benefit from these strategies.

## Figures and Tables

**Table 1:** Baseline characteristics by treatment strategy.

Variable	Overall (n = 173)	Monotherapy (n = 111)	Combination (n = 62)	Standardized Mean Difference (95% CI)		*p*
**Demographic**						
Age, median (IQR)	66 (54–73)	68 (59–75)	63 (52–71)	0.46		**0.008**
Male sex	123 (71.1)	78 (70.3)	45 (72.6)	0.04		0.748
**Comorbidity, n (%)**						
Cerebrovascular disease	37 (21.4)	28 (25.2)	9 (14.5)	0.21		0.099
Cardiovascular disease	49 (28.3)	34 (30.6)	15 (24.2)	0.12		0.312
Diabetes mellitus	36 (20.8)	24 (21.6)	12 (19.4)	0.04		0.724
COPD	37 (21.4)	25 (22.5)	12 (19.4)	0.06		0.626
CKD	33 (19.1)	25 (22.5)	8 (12.9)	0.20		0.122
Liver disease	10 (5.8)	7 (6.3)	3 (4.8)	0.05		0.692
Solid tumor	30 (17.3)	18 (16.2)	12 (19.4)	−0.06		0.601
Hematologic malignancy	5 (2.9)	3 (2.7)	2 (3.2)	−0.02		1.000
Immunosuppression	30 (17.3)	15 (13.5)	15 (24.2)	−0.23		0.075
**CCI ≥ 3, n (%)**	117 (67.6)	79 (71.2)	38 (61.3)	0.17		0.183
**Previous healthcare exposures, n (%)**						
Previous antibiotics	127 (73.4)	96 (86.5)	31 (50.0)	0.74		**<0.0001**
Previous hospitalization	75 (43.4)	52 (46.8)	23 (37.1)	0.16		0.214
**Indwelling invasive devices, n (%)**						
Vascular devices	156 (90.2)	100 (90.1)	56 (90.3)	0.00		0.961
Urinary devices	144 (83.2)	97 (87.4)	47 (75.8)	0.26		**0.050**
Respiratory/airway devices	47 (27.2)	20 (18.0)	27 (43.6)	−0.49		**0.0003**
Enteral/nutritional devices	8 (4.6)	5 (4.5)	3 (4.8)	−0.01		1.000
Drains/shunts	5 (2.9)	4 (3.6)	1 (1.6)	0.10		0.656
ECMO/CVVH	28 (16.2)	20 (18.0)	8 (12.9)	0.11		0.381
**Severity of illness, n (%)**						
ICU at time of infection onset	107 (61.8)	73 (65.8)	34 (54.8)	0.19		0.156
**Sepsis**						0.076
No	47 (27.2)	24 (21.6)	23 (37.1)	−0.29		
Sepsis	91 (52.6)	63 (56.8)	28 (45.2)	0.19		
Septic shock	35 (20.2)	24 (21.6)	11 (17.7)	0.08		
Sepsis plus Septic shock	126 (72.8)	87 (78.4)	39 (62.9)	0.29	0.29	**0.028**
**Infection type, n (%)**						0.179
Pneumonia	128 (74.0)	77 (69.4)	51 (82.3)	−0.24		
BSI	24 (13.9)	18 (16.2)	6 (9.7)	0.15		
Pneumonia + BSI	21 (12.1)	16 (14.4)	5 (8.1)	0.16		
**Prior MDR *P. aeruginosa* colonization, n (%)**	55 (31.8)	38 (34.2)	17 (27.4)	0.12		0.356
**Polymicrobial infection, n (%)**	29 (16.8)	21 (18.9)	8 (12.9)	0.13		0.310
**Novel β-lactams, n (%)**						**<0.0001**
CT	112 (64.7)	84 (75.7)	28 (45.2)	0.55		
CZA	35 (20.2)	8 (7.2)	27 (43.6)	−0.83		
Cefiderocol	15 (8.7)	10 (9.0)	5 (8.1)	0.03		
M/V	9 (5.2)	7 (6.3)	2 (3.2)	0.11		
IR	2 (1.2)	2 (1.8)	0	0.14		
**Type of combination therapy with:**						
1 other active antimicrobial	-	-	43 (69.4)			-
Fosfomycin	-	-	19 (30.6)			-
Aminoglycoside	-	-	13 (21.0)			-
Colistin	-	-	9 (14.5)			-
Fluoroquinolone	-	-	2 (3.2)			-
≥2 Active antimicrobials	-	-	19 (30.6)			-
**Infusion strategy, n (%)**						**0.0059**
Continuous/prolonged infusion	120 (69.4)	85 (76.6)	35 (56.4)	0.37		
Standard infusion	53 (30.6)	26 (23.4)	27 (43.6)	−0.30		
**Duration of treatment,** days, median (IQR)	12 (8–16)	10 (7–14)	15 (11–18)	−0.63		**0.0001**
**Length of stay**, days, median (IQR) *	64 (34–104)	66 (34–113)	66 (34–83)	0.10		0.551
**Outcomes, n (%)**						0.595
Clinical cure	112 (64.7)	73 (65.8)	39 (62.9)	0.05		
Microbiological recurrence within 30 days	27 (15.6)	16 (14.4)	11 (17.7)	−0.07		
Death	29 (16.8)	20 (18.0)	9 (14.5)	0.08		
**28-day mortality**	41 (23.7)	28 (25.2)	13 (21.0)	0.08		0.477 **

Abbreviations: IQR, interquartile range; COPD, chronic obstructive pulmonary disease; CKD, chronic kidney disease; CCI, Charlson comorbidity index; ECMO/CVVH, extracorporeal membrane oxygenation/continuous venovenous hemofiltration; ICU intensive care unit; MDR, multidrug-resistant; BSI, bloodstream infection; CT, ceftolozane–tazobactam; CZA, ceftazidime–avibactam; M/V; meropenem–vaborbactam; IR; imipenem–relebactam. * 19 missing values ** log-rank P. Bold text indicates the main variable categories; bold *p* values indicate statistical significance (*p* < 0.05).

**Table 2:** Univariate Cox regression analysis for 28-day mortality.

Variable	HR	95% CI	*p*
Age (per 1-year increase)	1.031	1.004–1.059	**0.024**
Cardiovascular disease, ref. N	1.952	1.042–3.658	**0.037**
Chronic renal disease, ref. N	1.573	0.833–2.970	0.163
ICU admission, ref. N	3.247	1.439–7.327	**0.005**
Sepsis, ref. N	5.003	1.514–16.534	**0.008**
Septic shock, ref. N	6.220	1.755–22.047	**0.005**
Treatment duration (per 1-day increase)	0.869	0.806–0.937	**0.0003**
Combination therapy, ref. monotherapy	0.789	0.409–1.523	0.480
Continuous/prolonged infusion, ref. standard	1.44	0.70–2.93	0.320

Abbreviations: CI, confidence interval; HR, hazard ratio; ICU, intensive care unit; N, no; ref., reference category. Bold *p* values indicate statistical significance (*p* < 0.05).

**Table 3:** Multivariable Cox regression analysis for 28-day mortality: all variables included in the model are reported.

Variable	Died n = 41	Survived n = 132	aHR	95% CI	*p*
Age (per 1-year increase)	-	-	1.025	0.994–1.058	0.117
Cardiovascular disease, ref. N	16	31	1.812	0.942–3.485	0.075
Chronic renal disease, ref. N	15	32	1.063	0.540–2.094	0.860
Sepsis, ref. N	26	65	3.202	0.898–11.410	0.073
Septic shock, ref. N	12	23	3.576	0.920–13.904	0.066
ICU admission, ref. N	34	73	2.395	1.004–5.713	**0.** **049**
ICU admission- no sepsis *	2	9	6.023	0.542–66.976	0.144
No ICU admission—sepsis/septic shock *	6	24	6.121	0.725–51.700	0.096
ICU admission—sepsis/septic shock *	32	64	12.950	1.741–96.340	**0.012**
Combination therapy, ref. N	13	49	1.095	0.593–2.207	0.800
Prolonged/Continuous infusion, ref. standard	31	89	0.918	0.424–1.987	0.827

Abbreviations: CI, confidence interval; HR, hazard ratio; ICU, intensive care unit; N, no; ref., reference category. * The aHRs were calculated using alternative ICU admission and presence of sepsis/septic shock or the combined variable. Bold *p* values indicate statistical significance (*p* < 0.05).

## Data Availability

The data that support the findings of this study are available from the corresponding author, upon reasonable request.

## Supplementary Materials

The following supporting information can be downloaded at https://www.mdpi.com/article/10.3390/antibiotics15080796/s1: Table S1: Baseline characteristics of study population by infusion strategy; Table S2: Co-isolated microorganisms in polymicrobial infections.

## Abbreviations

The following abbreviations are used in this manuscript:

aHR	Adjusted hazard ratio
AIDS	Acquired immunodeficiency syndrome
BL/BLI	β-lactam/β-lactamase inhibitor
BSI	Bloodstream infection
CCI	Charlson Comorbidity Index
CI	Confidence interval
COPD	Chronic obstructive pulmonary disease
CR	Carbapenem-resistant
C/T	Ceftolozane/tazobactam
CZA	Ceftazidime/avibactam
CVVH	Continuous venovenous hemofiltration
ECMO	Extracorporeal membrane oxygenation
HAP	Hospital-acquired pneumonia
HR	Hazard ratio
ICU	ICU
IQR	Interquartile range
IR	Imipenem–relebactam
MDR	Multidrug-resistant
MIC	Minimum inhibitory concentration
PK/PD	Pharmacokinetic/pharmacodynamic
RRT	Renal Replacement therapy
SUSANA	SUrveillance of SAfety and outcome of New Antibiotics
TDM	Therapeutic drug monitoring
VAP	Ventilator-associated pneumonia
