# Supplementary material for: Real-World Antibiotic Strategies for Multidrug-Resistant Pseudomonas aeruginosa: Evidence from the Italian SUSANA Multicentre Study
Source: Antibiotics (Basel). 2026 Aug 17;15(8):796. doi: 10.3390/antibiotics15080796 (PMC13509685; doi:10.3390/antibiotics15080796)
Supplement: Supplementary file 1 [file antibiotics-15-00796-s001.zip › antibiotics-4429652-supplementary.pdf]

antibiotics

Supplementary Tables

Supplementary Table S1. Baseline characteristics of study population by infusion strategy.

Variable	Overall n=173	Continuous/Prolonged n=120	Standard n=53	p-value
**Demographic**				
Age, median [IQR]	66 [54–73]	67.5 [55.0–73.0]	65.0 [54.0–72.0]	0.3898
Male sex	123 (71.1)	89 (74.2)	34 (64.2)	0.1804
**Comorbidity, n (%)**				
Cerebrovascular disease	37 (21.4)	26 (21.7)	11 (20.8)	0.8927
Cardiovascular disease	49 (28.3)	39 (32.5)	10 (18.9)	0.1729
Diabetes mellitus	36 (20.8)	23 (19.2)	13 (24.5)	0.4232
COPD	37 (21.4)	31 (25.8)	6 (11.3)	**0.0319**
CKD	33 (19.1)	21 (17.5)	12 (22.6)	0.4275
Liver disease	10 (5.9)	7 (4.1)	3 (1.7)	0.9642
Solid tumor	30 (17.3)	17 (14.2)	13 (24.5)	0.0970
Hematologic malignancy	5 (2.9)	4 (3.3)	1 (1.9)	0.6006
Immunodepression	30 (17.3)	14 (11.7)	16 (30.2)	**0.0030**
**CCI ≥3**	117 (67.6)	81 67.5)	36 (67.9)	0.9561
**Inderwelling invasive devices, n (%)**				
Vascular device	156 (90.2)	113 94.2)	43 (81.1)	**0.0079**
Urinary device	144 (83.2)	106 88.3)	38 (71.7)	**0.0069**
Respiratory/airway device	47 (27.2)	33 27.5)	14 (26.4)	0.8824
Enteral/nutritional devices	8 (4.6)	6 5.0)	2 (3.8)	0.7233
Drainage/shunt device	5 (2.9)	3 2.5)	2 (3.8)	0.6448
ECMO/CVVH	28 (16.2)	22 18.3)	6 (11.3)	0.2483
**Severity of illness, n (%)**				
ICU ward at infection onset	107 (61.9)	82 68.3)	25 (47.2)	**0.0290**
**Sepsis**				
No	47 (27.2)	20 16.7)	27 (50.9)	**<0.0001**
Sepsis	91 (52.6)	73 60.8)	18 (34.0)	
Septic shock	35 (20.2)	27 22.5)	8 (15.1)	
**Infection type, n (%)**				
Pneumonia	128 (74.0)	89 (74.2)	39 (73.6)	
BSI	24 (13.9)	19 15.8)	5 (9.4)	
Pneumonia + BSI	21 (12.1)	12 10.0)	9 (17.0)	
**Polymicrobial infection, n (%)**	29 (16.8)	23 (19.2)	6 (11.3)	0.203
**Novel β-lactams, n (%)**				
CT	112 (64.7)	79 65.8)	33 (62.3)	0.0762
CZA	35 (20.2)	21 17.5)	14 (26.4)	
Cefiderocol	15 (8.7)	12 10.0)	3 (5.7)	
M/V	9 (5.2)	8 6.7)	1 (1.9)	
IR	2 (1.2)	0 0.0)	2 (3.8)	
**Treatment strategy, n (%)**				

Variable	Overall n=173	Continuous/Prolonged n=120	Standard n=53	p-value
Combination therapy	48 (27.7)	30 25.0)	18 (34.0)	0.2249
Monotherapy	125 (72.3)	90 75.0)	35 (66.0)	
**Duration of treatment, days, median (IQR)**	—	11.0 [7.0–15.0]	13.0 [9.0–16.0]	0.1797
**Hospital stay, median IQR**	—	68.0 [37.0–111.0]	51.0 [26.0–90.0]	0.1943
**Outcomes, n (%)**				
Clinical cure	112 (64.7)	79 65.8)	33 (62.3)	0.8191
Microbiological recurrence within 30 days	27 (15.6)	17 14.2)	10 (18.9)	
28-day mortality	41 (23.7)	31 25.8)	10 (18.9)	0.3206

Abbreviations: IQR, interquartile range; COPD, chronic obstructive pulmonary disease; CKD, chronic kidney disease; CCI, Charlson comorbidity index; ECMO/CVVH, extracorporeal membrane oxygenation/continuous venovenous hemofiltration; ICU intensive care unit; MDR, multidrug-resistant resistant; BSI, blood stream infection; CT, ceftolozane-tazobactam; CZA, ceftazidime-avibactam; M/V; meropenem-vaborbactam; IR; imipenem-relebactam

Supplementary Table S2. Co-isolated microorganisms in polymicrobial infections.

Co-pathogen in polymicrobial episodes (N=29)	Episodes, n (%)
Klebsiella pneumoniae	14 (48.3)
Enterococcus faecium	4 (13.8)
Serratia marcescens	4 (13.8)
Acinetobacter baumannii	3 (10.3)
Escherichia coli	2 (6.9)
Staphylococcus aureus	2 (6.9)
Stenotrophomonas maltophilia	2 (6.9)
Achromobacter xylosoxidans	1 (3.4)
Staphylococcus epidermidis	1 (3.4)
Delftia acidovorans	1 (3.4)

Percentages are calculated among polymicrobial infections. Co-isolated microorganisms were counted once per episode; totals may exceed 100% because more than one co-pathogen could be isolated in the same episode.
